# Validation of an instrument to assess informal caregivers’ perceptions about the delivery of patient-centred care to people with intellectual disabilities in residential settings

**DOI:** 10.1186/s12913-019-4358-9

**Published:** 2019-07-24

**Authors:** Jane Murray Cramm, Anna Petra Nieboer

**Affiliations:** Erasmus School of Health Policy and Management, Department of Socio-Medical Sciences, Erasmus University Rotterdam, Rotterdam, The Netherlands

**Keywords:** Patient centred care, Intellectual disability, Disability organisation, Instrument development, Informal caregiver, PCC-IC

## Abstract

**Background:**

Validated instruments are needed to assess the delivery of patient-centred care (PCC) to people with intellectual disabilities (PWIDs) needing 24-h care in residential settings. Eight dimensions of PCC have been identified: taking patients’ preferences into account; access to care; emotional support; physical comfort; information and education; involvement of family and friends; coordination of care; and continuity and secure transition. Objective of this study is to validate an instrument to assess these eight PCC dimensions among informal caregivers of PWIDs in residential settings (institutional settings as well as group homes in the community). The original 24-item instrument was developed and validated among professionals providing care to PWIDs.

**Methods:**

This study was conducted in a disability care centre in the Netherlands. All informal caregivers of PWIDs living in institutional settings or group homes in the community in need of 24-h care were invited to participate (*n* = 941). The response rate was 31% (*n* = 289). We tested the instrument using structural equation modelling, and examined its validity and reliability.

**Results:**

Confirmatory factor analyses revealed good indices of fit and overall internal consistency, as represented by Cronbach’s alpha values. All eight dimensions of PCC were related positively to satisfaction with care (all *p* ≤ 0.001). As expected, informal caregivers were less critical of PCC and its underlying dimensions, except for information and education, than were professionals working in the same disability care centre.

**Conclusions:**

The psychometric properties of the 24-item PCC instrument for informal caregivers (PCC-IC) were satisfactory, indicating that the PCC-IC is valid and reliable for the assessment of the eight dimensions of PCC among informal caregivers of PWIDs in residential settings.

**Electronic supplementary material:**

The online version of this article (10.1186/s12913-019-4358-9) contains supplementary material, which is available to authorized users.

## Background

Since the Institute of Medicine identified patient/person-centred care (PCC) as one of its six quality improvement domains the importance of this subject has grown tremendously. A commonly used definition of PCC is: ‘healthcare that establishes a partnership among practitioners, patients, and their families (when appropriate) to ensure that decisions respect patients’ wants, needs, and preferences and that patients have the education and support they need to make decisions and participate in their own care’ [1]. Years of research has led to the identification of eight PCC dimensions: taking patients’ preferences into account; access to care; emotional support; physical comfort; information and education; involvement of family and friends; coordination of care; and continuity and secure transition [2–11]. A systematic review [9] clearly showed that organizations who do better in terms of the eight PCC dimensions also report better patient and organizational outcomes. However, the systematic review included mainly studies conducted in hospital settings and some in the primary care setting, relevant research for people with intellectual disabilities (PWIDs) is lacking. There are studies showing the importance of person centred care planning in which PWIDs and their informal caregivers are given a more important role in the care planning process [12, 13]. But the care planning process is only one of the eight PCC dimensions and a more in-depth understanding is needed of all eight dimensions in residential settings. In order to study these eight dimensions of PCC for PWIDs we need a valid instrument to asses these aspects among informal caregivers. Only recently a valid and reliable instrument was developed to assess the eight dimensions of PCC for professionals providing care for PWIDs [14], but no instrument for informal caregivers of PWIDs is currently available. Informal caregivers are usually close family members (mainly parents and siblings) who often are crucial links between PWIDs and formal care providers, as they can secure the establishment of individualised care. The study of Maaskant and Hoekman [15] shows that although around 50% of PWIDs in the Netherlands live in residential care facilities, placement in a long-term care facility does not reduce caregiver burden. When parents of PWIDs no longer co-reside with their adult child, they generally still remain very involved in their care delivery [16] and many of the parents continue to play an active role by providing assistance to meet their child’s daily care needs [17]. Given these caregivers’ prominent roles in care delivery, the development and validation of measures for the assessment of PCC provided to PWIDs from their perspective is needed. Therefore, the aim of this study is to validate the PCC instrument designed for professionals providing care to PWIDs among informal caregivers of PWIDs. Items were adjusted to assess informal caregivers’ assessment of PCC provided to PWIDs living in a residential setting. We conducted psychometric testing to determine the validity and reliability of the PCC instrument when applied to these caregivers.

Given that experiences with care are known to differ between professionals and informal caregivers [e.g.18, 19], we compared scores from these groups as part of the validation. Based on previous research [18, 19], we expected that professionals would be more critical than informal caregivers in reporting on their experiences when it comes to PWIDs in residential settings. Professionals work with various clients in diverse situations, and even highly trained and experienced professionals are not always able to deliver the care they aim to give. In contrast, informal caregivers experience care delivery to single specific persons. Furthermore, research clearly shows that enhanced levels of the eight PCC dimensions are associated positively with patients’ satisfaction with care [9] and professionals’ satisfaction with work [14, 20]. Given the close relationship and involvement of informal caregivers in the care delivery process, we expected that investment in the eight dimensions of PCC would also be associated positively with informal caregivers’ satisfaction with care.

## Methods

### Setting and participants

A cross-sectional survey was conducted in a disability care centre called the Twentse Zorgcentra in the eastern part of the Netherlands. The organization provides mainly residential care with 4 to 8 PWIDs who live together in both institutional settings and community group homes requiring 24-h care. Informal caregivers of all PWIDs living in these residential settings (*n* = 941) were invited to participate. Data were collected in April–June 2015 using postal questionnaires. After 1 postal reminder a total of 289 (31% response rate) informal caregivers responded to this survey.

According to the national Dutch guidelines carried out by the Central Committee on Research Involving Human Subjects (the national ethics committee in the Netherlands), this study did not fall within the scope of the Medical Research Involving Human Subjects Act. We investigated informal caregivers’ perceptions not patients. It was thus exempted from review by an accredited medical research and ethics committee or the CCMO. All respondents were informed about the study aims and its anonymous and voluntary nature before they consented to participate. By filling in the questionnaire and sending them back to us consent was implied.

### Survey measures

#### PCC

The eight dimensions of PCC, as identified by the Picker Institute, were used as a framework for the development of 24 items for the assessment of PCC for PWIDs. The current instrument for informal caregivers was based on a version tested on professionals providing care for PWIDs [14]. Items for both the professionals and informal caregiver version were developed in close collaboration with experts in the field. The questionnaire was approved by the client council which mainly consists of informal caregivers. They agreed that the content of the items were relevant and interesting to investigate. They thought no further adjustments were needed. The 24-item version for informal caregivers (PCC-IC) is provided in Additional file 1. Respondents were asked about the level of PCC provided to their loved one within the ‘Twentse Zorgcentra’ during the past 4 months. Item responses were structured by a 5-point scale ranging from 1 (never) to 5 (always), with higher scores indicating better PCC.

#### Satisfaction with care

Satisfaction with care was assessed using an adjusted version of the Caregivers’ Satisfaction with inpatient Stroke Care (C-SASC; see Additional file 2). Responses to the seven items were structured by a 4-point scale ranging from totally disagree to totally agree. This instrument was developed and validated in the Netherlands and has shown high degrees of reliability and construct validity [21]. Although the SASC (for patients) and C-SASC (for caregivers) were originally developed for stroke patients, they have been used widely in various patient populations to assess satisfaction with care in general [e.g. 22–26]. The items were slightly adjusted and those less relevant were removed from the questionnaire, resulting in a final set of 7 items to assess satisfaction of care among informal caregivers of institutionalized PWIDs: ‘I have been treated with kindness and respect by the staff’, ‘The staff attended well to my personal needs and tried to support me as much as possible’, ‘I was able to talk to the staff about any problems I might have had’, ‘I have received all the information I want about the nature of the disability of the person I take care of’, ‘The staff did everything they can to improve the situation for the person I take care of’, ‘I am satisfied with the type of treatment the therapists have given the person I take care of (e.g., personal guidance, physiotherapy, speech therapy, occupational therapy)’ and ‘The person I take care of has been treated with kindness and respect by the staff’. The Cronbach’s alpha value of the C-SASC in this study was 0.88, indicating good reliability.

#### Background characteristics

The survey contained questions about informal caregivers’ demographic characteristics age, gender, marital status, educational level, and working hours/week. Dummy variables were created for marital status (married /living with partner (0) - living alone, widowed or divorced (1), education (low = primary education or less; medium = prep school for vocational secondary education or secondary vocational education; high = senior general secondary education, pre-university education, higher professional education or university). In addition, questions were asked about relationships to care recipients, time spent providing informal care (hours/week) and duration of care (years). Dummy variables were created for time spent caring in hours per week (less than 8 h (0) - ≥ 8 h (1)), years providing informal care (less than 10 years (0) - ≥ 10 years (1)).

### Analysis

Our analysis involved the following six steps.

1. Descriptive statistics were used to characterise the study population.
2. For each PCC item, the number of missing responses and the mean and standard deviation were determined.
3. Confirmatory factor analysis was performed to verify the factor structure of the 24-item questionnaire using LISREL [22]. We treated the data as ordinal and used robust diagonally weighted least squares (DWLS) estimation with polychoric correlations to fit factor models. The robust DWLS method has been recommended by others [23] for ordinal data with five or fewer categories.
4. Multiple imputation techniques (expected maximisation algorithm) were used to test the measurement model. Six respondents were excluded because they did not respond to any of the PCC questions, resulting in a final study sample of 283 informal caregivers. The following indices of model fit, with cut-off criteria proposed by Hu and Bentler [24] and Steiger [25], were used:

- the standardised root mean square residual (SRMR), a scale-invariant global fit index ranging from 0 to 1 (SRMR < 0.08 indicates good fit);
- the root mean square error of approximation (RMSEA), which according to Steiger [25] should be close to 0.07; and
- the comparative fit index (CFI), which compares the independent (i.e. observed variables are unrelated) and estimated models and should exceed 0.95.

5. The internal consistency of the subscales was assessed using Cronbach’s alpha. Inter-correlations were investigated to verify conceptual relatedness among (sub)scales. We also computed a composite reliability index based on the factor loadings of the first-order constructs to assess overall scale reliability.
6. The construct validity of the PCC instrument (overall and the eight dimensions) was assessed by analysing associations with satisfaction with care, using list-wise deletion of missing cases. Finally, we compared the mean PCC scores of informal caregivers and professionals working in the same institution. Professionals who had worked for the organisation for at least 1 year and working for at least 16 h of work per week, were also selected to fill in a questionnaire (*n* = 1146) of which 466 (40%) responded [14]. Two respondents only filled in background characteristics and therefore these two respondents were eliminated from the analyses bringing the total n to 464.

## Results

### Informal caregiver characteristics

A total of 289 respondents filled in the questionnaire (response rate of 31%). The mean age of the informal caregivers was 61.51 ± 11.13 (range 23–90) years (Table 1). About half (57%) of respondents were female and 23% were single. Most (83%) respondents had provided informal care for > 10 years and 30% provided ≥8 h informal care per week. About half of the respondents (46%) were parents providing informal care to their children and a total of 44% were siblings providing informal care to their brother/sister. The remaining respondents were more distant family members (e.g. grandchildren, grandparents, cousins). All informal caregivers cared for institutionalised clients. The level of required care and support, however, differed. Almost one-third (31%) of informal caregivers’ clients required intensive care and support, and 60% of clients had such severe conditions that they required highly intensive support. The remaining 9% of informal caregivers’ clients needed (some) care and support.

**Table 1:** Characteristics of informal caregivers (*n* = 289)

Characteristic	Mean (standard deviation) range or percentage
Age (years)	61.51 (11.13) 23–90
Gender (female)	56.8%
Education	
Low	10.0%
Medium	64.0%
High	26.0%
Marital status (single)	23.4%
Time spent caring (≥8 h/week)	30.3%
Years caring (≥10)	82.5%
Person-centred care score	3.76 (0.67) 1–5
Satisfaction with care score	3.46 (0.44) 1–4

### PCC item characteristics

Mean scores for all items in the patient preferences and access to care dimensions exceeded 4.0 (Table 2). Mean scores for items in the information and education dimension were lowest.

**Table 2:** Characteristics of the 24 person-centred care items (*n* = 289)

	Item	Valid *n*	Missing	Mean	SD	λ
*Patients’ preferences*						
1.	Healthcare professionals treat clients with dignity and respect	285	4 (1%)	4.56	0.69	0.820
2.	Healthcare is focused on improving the quality of life of clients	286	4 (1%)	4.30	0.77	0.882
3.	Healthcare professionals take clients’ preferences into account	286	4 (1%)	4.08	0.83	0.885
*Physical comfort*						
6.	Healthcare professionals pay attention to pain management	271	18 (6%)	3.93	1.07	0.733
7.	Healthcare professionals take clients’ preferences for support with their daily living needs into account	280	9 (3%)	4.05	1.00	0.740
9.	Clients have privacy	281	8 (3%)	3.78	1.04	0.575
*Coordination of care*						
10.	Healthcare professionals are well informed; clients need to tell their story only once	264	25 (9%)	3.50	1.10	0.797
11.	Care is well coordinated among professionals	282	7 (2%)	3.76	0.93	0.785
14.	Healthcare professionals work as a team in care delivery to clients	282	7 (2%)	4.20	0.90	0.661
*Emotional support*						
15.	Healthcare professionals pay attention to clients’ anxiety about their situations	270	19 (7%)	4.02	1.02	0.903
16.	Healthcare professionals involve relatives in the emotional support of clients	274	15 (5%)	4.01	1.06	0.836
17.	Healthcare professionals pay attention to clients’ anxiety over the impact of their illness on their loved ones (if applicable)	246	43 (15%)	3.68	1.21	0.851
*Access to care*						
18.	The building is accessible to all clients	279	10 (3%)	4.43	0.98	0.537
19.	Clear directions are provided to and inside the building	266	23 (8%)	4.28	1.00	0.595
20.	It is easy to schedule an appointment	284	5 (2%)	4.30	0.92	0.825
*Continuity and transition*						
23.	When a client is transferred to another ward, relevant patient information is transferred as well	264	25 (9%)	4.06	1.17	0.759
24.	Clients who are transferred are well informed about where they are going, what care they will receive, and who will be their contact person	262	27 (9%)	3.89	0.67	0.918
25.	Clients get skilled advice about care and support at home after discharge	222	67 (23%)	3.87	1.27	0.976
*Information and education*						
27.	Clients can access their care records	227	62 (21%)	2.47	1.66	0.701
28.	Clients are in charge of their own care	237	52 (18%)	2.59	1.42	0.953
29.	Healthcare professionals support clients to be in charge of their care	253	36 (12%)	3.19	1.33	0.923
*Family and friends*						
33.	Healthcare professionals involve relatives in decisions regarding patients’ care	281	8 (3%)	4.38	0.93	0.786
34.	Healthcare professionals pay attention to loved ones in their role as carers for clients	279	10 (3%)	4.01	1.00	0.870
35.	Healthcare professionals pay attention to the needs of clients’ family and friends	280	9 (3%)	3.79	1.09	0.857

Item non-response rates ranged from 1 to 23%. The most problematic items were ‘clients get skilled advice about care and support at home after discharge’ (23% missing responses), ‘clients can access their care records’ (21% missing responses), and ‘clients are in charge of their own care’ (18% missing responses).

### Fit and factor loading

The model showed good fit, meeting cut-off criteria (CFI = 0.989, SRMR = 0.0567, RMSEA = 0.0560). All items had factor loadings > 0.50 on the intended factors (Table 2). In addition, we tested a second-order factor structure. The second-order solution also showed good model fit (CFI = 0.986, RMSEA = 0.061, SRMR = 0.0639) and all loadings of the second-order factor were > 0.50 (all *p* < 0.001). We did not formally compare the models using a χ^2^ difference test, because χ^2^ fit statistics and the derived difference test are highly sensitive to sample size. Rather, we compared the alternative goodness-of-fit indices RMSEA, CFI and SRMR. The results were comparable, although the RMSEA value was higher for the second-order model.

### Internal consistency and inter-correlations

Internal consistency values for the subscales ranged from 0.61 (access to care) to 0.86 (emotional support and continuity and transition; Table 3). The internal consistency value for the 24-item PCC instrument (a composite reliability index based on the factor loadings of the first-order constructs) was 0.956. All (sub)scales were significantly and positively correlated (all *p* ≤ 0.001), indicating that they were conceptually related.

**Table 3:** Scale characteristics and (inter-)correlations of the 24-item person-centred care instrument

	Cronbach’s *α*	Scale mean (SD)	1	2	3	4	5	6	7	8
1. Patients’ preferences	0.82	4.31 (0.66)								
2. Physical comfort	0.65	3.92 (0.80)	0.69***							
3. Coordination of care	0.73	3.83 (0.79)	0.70***	0.71***						
4. Emotional support	0.86	3.91 (0.96)	0.67***	0.66***	0.66***					
5. Access to care	0.61	4.34 (0.72)	0.48***	0.45***	0.47***	0.46***				
6. Continuity and transition	0.86	3.95 (0.89)	0.60***	0.60***	0.66***	0.60***	0.56***			
7. Information and education	0.85	2.75 (1.30)	0.34***	0.39***	0.45***	0.34***	0.29***	0.47***		
8. Family and friends	0.82	4.06 (0.86)	0.56***	0.55***	0.59***	0.66***	0.43***	0.54***	0.31***	
9. Overall PCC	0.96^a^	3.88 (0.67)	0.81***	0.82***	0.84***	0.82***	0.67***	0.83***	0.64***	0.75***

Notes: *PCC*, Person-centred care. ***p < 0.001 (two-tailed). Results are based on list-wise deletion of missing cases. ^a^ Composite reliability index based on the factor loadings of the first-order construct

### Construct validity

All eight dimensions of PCC were related positively to satisfaction with care (all *p* ≤ 0.001; Table 4), indicating construct validity. Strongest relationships were found between satisfaction with care and the following four PCC dimensions: family and friends (*r* = 0.58), coordination of care (*r* = 0.56), patient preferences (*r* = 0.52), and physical comfort (*r* = 0.51).

**Table 4:** Correlations of person-centred care dimensions with satisfaction with care

	Satisfaction with care
Patients’ preferences	0.52***
Physical comfort	0.51***
Coordination of care	0.56***
Emotional support	0.47***
Access to care	0.38***
Continuity and transition	0.44***
Information and education	0.35***
Family and friends	0.58***
Overall person-centred care	0.62***

Notes: ***p < 0.001 (two-tailed). Results are based on list-wise deletion of missing cases

### Comparison of professionals’ and informal caregivers’ PCC scores

Table 5 displays the mean scores given by professionals providing care to PWIDs in the Twentse Zorgcentra and by informal caregivers of institutionalised PWIDs. In general, professionals were more critical than informal caregivers (as indicated by lower scores on seven of the eight dimensions and their overall perceptions of PCC). Information and education was the only dimension in which informal caregivers were less satisfied than professionals.

**Table 5:** Comparison of PCC scores between professionals and informal caregivers

PCC dimension	Mean score, professionals	Mean score, informal caregivers	*p*-value
Patients’ preferences	4.05 (0.58)	4.32 (0.66)	< 0.001
Physical comfort	3.57 (0.73)	3.92 (0.80)	< 0.001
Coordination of care	3.64 (0.67)	3.83 (0.79)	< 0.001
Emotional support	3.64 (0.83)	3.91 (0.96)	< 0.001
Access to care	3.50 (0.81)	4.34 (0.72)	< 0.001
Continuity and transition	3.44 (0.84)	3.95 (0.89)	< 0.001
Information and education	3.08 (0.98)	2.75 (1.30)	< 0.001
Family and friends	3.89 (0.80)	4.06 (0.86)	< 0.001
Overall PCC	3.60 (0.55)	3.88 (0.68)	< 0.001

Notes: *PCC*, Person-centred care. The results of the professionals are based on a sample of *N* = 464

## Discussion

The results of this study provided preliminary evidence supporting the validity and reliability of the 24-item PCC-IC instrument to assess PCC and its eight dimensions from the perspective of informal caregivers of PWIDs. Items under “information and education” and “continuity and transition” contained large numbers of missing data. Although a large number of respondents thought they were not applicable, for those who did think they were applicable these dimensions were, however, relevant. More research is needed to investigate the importance of these dimensions across groups.

Results of this study also showed that the advantages achieved by healthcare organisations delivering high-level PCC are likely to enhance satisfaction with care among these caregivers. Strong correlations were observed with various PCC dimensions. Investment in the eight PCC dimensions is known to lead to better patient and organisational outcomes [9]; this study adds to this knowledge and shows that the patient preferences, physical comfort, coordination of care, and family and friends dimensions of PCC especially enhanced informal caregivers’ satisfaction with care delivery. This information is important for those aiming to improve levels of patient-centredness and satisfaction with care in their organisations. Primary barriers to the improvement of PCC are the lack of focus on it as a quality indicator and the untimely conduction of performance reports [26]. Gathering information about ‘real’ experiences with PCC (instead of objective quality indicators) on a regular basis is crucial for evaluation and has been shown to be the best approach to PCC measurement [27].

This research also showed differences in experiences between professionals providing care to PWIDs and informal caregivers for this population, in line with previous research [18, 19]. Informal caregivers were more positive about overall PCC and the patient preferences, physical comfort, coordination of care, emotional support, access to care, continuity and transition, and family and friends dimensions. When filling in the questionnaire, informal caregivers had their personal, individual experiences in mind, whereas professionals were thinking of care delivery to all PWIDs in general. Professionals will more regularly notice problems with care coordination, transition, and continuity, for example, than will informal caregivers. Informal caregivers were more negative than professionals about the information and education dimension of PCC. We selected informal caregivers of institutionalised PWIDs only, excluding, for example, those providing only day care. For some of these clients, information and education are not relevant because of low levels of cognitive functioning, which likely affected informal caregivers’ responses. In contrast, professionals answered these questions with the entire population in mind, which may explain the difference in experience.

This study has several limitations. First and most importantly, we did not examine the perceptions of PWIDs. Further research is necessary to develop and validate an instrument for the assessment of patient-centeredness in organisations from PWIDs’ perspective. Also, convergent validity could be strengthened by applying other instruments and methods (e.g. by using other instruments to correlate the instrument with or by using other methods such as observing client-staff interactions). Second, we did not examine the predictive value of the 24-item PCC-IC instrument. Further research assessing the instrument’s sensitivity to change is needed. Third, this research revealed three items that were problematic for some respondents (> 15% missing responses): ‘clients get skilled advice about care and support at home after discharge’, ‘clients are in charge of their own care’, and ‘clients can access their care records’. Although these items are less relevant or simply not applicable to clients with severe intellectual disabilities, they are important in terms of PCC for clients with higher IQ levels. We resolved this issue by using multiple imputation techniques. Fourth, we were not able to match experiences of professionals and informal caregivers at the client level. Future research investigating the experiences of professionals and informal caregivers who have the same clients in mind would provide more detailed information on how their experiences differ and on which underlying PCC dimensions they (dis)agree. Such information would help organisations to identify potential discrepancies and find room for improvement. Fifth, the response of 31% may indicate non-response bias. We, however, do not know if the responders are those who were more or less positive about PCC within this organization. Sixth, we investigated informal caregivers of institutionalized PWIDs and those living in group homes in the community only. More research is needed among PWIDs living on their own in the community who are not in need of 24-h care. Finally, this instrument was developed in close collaboration with professionals and experts in the field of care for PWIDs, not with informal caregivers.

## Conclusions

This study showed that the psychometric properties of the 24-item PCC-IC instrument are good, and that the instrument can be used to assess the eight dimensions of PCC provided to PWIDs in residential settings from the perspective of informal caregivers. Previous research showed that this instrument is valid among professionals providing care to PWIDs [14]; the current study provided preliminary evidence supporting its validity and reliability among informal caregivers of PWIDs living in residential settings. Organisations aiming to improve PCC in this context could use the instrument to identify dimensions that should be targeted more directly for improvement through interventions. Given the difference in experience, we recommend assessment of the PCC dimensions among professionals and informal caregivers.

## Additional files

Additional file 1: The 24-item version for informal caregivers (PCC-IC). (DOCX 13 kb)

Additional file 2: Satisfaction with care questionnaire (adjusted Caregivers’ Satisfaction with inpatient Stroke Care). (DOCX 11 kb)

## Data Availability

The data and surveys used are available upon request (for those interested please email cramm@eshpm.eur.nl).

## Abbreviations

CFI: Comparative Fit Index
C-SASC: Caregivers’ Satisfaction with inpatient Stroke Care
DWLS: Diagonally Weighted Least Squares
PCC: Patient-Centred Care
PCC-IC: Patient-Centred Care instrument for Informal Caregivers
PWIDs: People With Intellectual Disabilities
RMSEA: Root Mean Square Error of Approximation
SRMR: Standardised Root Mean Square Residual
