# Supplementary material for: Validation of an instrument to assess informal caregivers’ perceptions about the delivery of patient-centred care to people with intellectual disabilities in residential settings
Source: BMC Health Serv Res. 2019 Jul 24;19:518. doi: 10.1186/s12913-019-4358-9 (PMC6657207; doi:10.1186/s12913-019-4358-9)
Supplement: Supplementary file 1 — The 24-item version for informal caregivers (PCC-IC). (DOCX 13 kb) [file 12913_2019_4358_MOESM1_ESM.docx]

**Additional file 1 PATIENT CENTRED CARE QUESTIONNAIRE**

| **PCC Dimensions** | **Questions** | **Scoring 1 - 5** |
| --- | --- | --- |
| Patients’ preferences | 1. Healthcare professionals treat clients with dignity and respect. | 5. always  4. often  3. regularly  2. sometimes  1. never |
|  | 2. Healthcare is focused on improving the quality of life of clients. | 5. always  4. often  3. regularly  2. sometimes  1. never |
|  | 3. Healthcare professionals take client's preferences into account. | 5. always  4. often  3. regularly  2. sometimes  1. never |
| Physical comfort | 4. Healthcare professionals pay attention to pain management. | 5. always  4. often  3. regularly  2. sometimes  1. never |
|  | 5. Healthcare professionals take client's preferences for support with their daily living needs into account. | 5. always  4. often  3. regularly  2. sometimes  1. never |
|  | 6. Clients have privacy. | 5. always  4. often  3. regularly  2. sometimes  1. never |
| Coordination of care | 7. Healthcare professionals are well-informed; clients need to tell their story only once. | 5. always  4. often  3. regularly  2. sometimes  1. never |
|  | 8. Care is well-coordinated between professionals. | 5. always  4. often  3. regularly  2. sometimes  1. never |
|  | 9. Healthcare professionals work as a team in care delivery to clients. | 5. always  4. often  3. regularly  2. sometimes  1. never |
| Emotional support | 10. Healthcare professionals pay attention to client's anxiety about their situation. | 5. always  4. often  3. regularly  2. sometimes  1. never |
|  | 11. Healthcare professionals involve relatives in the emotional support of the client. | 5. always  4. often  3. regularly  2. sometimes  1. never |
|  | 12. Healthcare professionals pay attention to client's anxiety over the impact of their illness on their loved ones. | 5. always  4. often  3. regularly  2. sometimes  1. never |
| Access to care | 13. The building is accessible to all clients. | 5. always  4. often  3. regularly  2. sometimes  1. never |
|  | 14. Clear directions are provided to and inside the building. | 5. always  4. often  3. regularly  2. sometimes  1. never |
|  | 15. It is easy to schedule an appointment. | 5. always  4. often  3. regularly  2. sometimes  1. never |
| Continuity and transition | 16. When a client is transferred to another ward, relevant patient information is transferred as well. | 5. always  4. often  3. regularly  2. sometimes  1. never |
|  | 17. Clients who are transferred are well-informed about where they are going, what care they will receive and who will be their contact person. | 5. always  4. often  3. regularly  2. sometimes  1. never |
|  | 18. Clients get skilled advice about care and support at home after discharge. | 5. always  4. often  3. regularly  2. sometimes  1. never |
| Information and education | 19. Clients can access their care records. | 5. always  4. often  3. regularly  2. sometimes  1. never |
|  | 20. Clients are in charge of their own care. | 5. always  4. often  3. regularly  2. sometimes  1. never |
|  | 21. Healthcare professionals support clients to be in charge of their care. | 5. always  4. often  3. regularly  2. sometimes  1. never |
| Family and friends | 22. Healthcare professionals involve relatives in decisions regarding the patient’s care. | 5. always  4. often  3. regularly  2. sometimes  1. never |
|  | 23. Healthcare professionals pay attention to loved ones in their role as carer for the client. | 5. always  4. often  3. regularly  2. sometimes  1. never |
|  | 24. Healthcare professionals pay attention to the needs of family and friends of the client. | 5. always  4. often  3. regularly  2. sometimes  1. never |
