# Supplementary material for: Validation of an instrument to assess informal caregivers’ perceptions about the delivery of patient-centred care to people with intellectual disabilities in residential settings
Source: BMC Health Serv Res. 2019 Jul 24;19:518. doi: 10.1186/s12913-019-4358-9 (PMC6657207; doi:10.1186/s12913-019-4358-9)
Supplement: Supplementary file 2 — Satisfaction with care questionnaire (adjusted Caregivers’ Satisfaction with inpatient Stroke Care). (DOCX 11 kb) [file 12913_2019_4358_MOESM2_ESM.docx]

**Additional File 2: Satisfaction with care questionnaire (adjusted Caregivers’ Satisfaction with inpatient Stroke Care)**

1. I have been treated with kindness and respect by the staff

2. The staff attended to my personal needs and tried to support me as much as possible

3. I was able to talk to the staff about any problems I might have had

4. I received all the information I wanted to about the nature of the disability of the person I take care of

5. The staff did everything they could to improve the situation of the person I take care of

6. I am satisfied with the type of treatment the therapists have given the person I take care of (e.g. giving personal support, speech therapy, occupational therapy)

7. The person I take care of has been treated with kindness and respect by the staff
